# Maternal Low-Protein Diet Modulates Glucose Metabolism and Hepatic MicroRNAs Expression in the Early Life of Offspring †

**DOI:** 10.3390/nu9030205

**Published:** 2017-02-27

**Authors:** Jia Zheng, Xinhua Xiao, Qian Zhang, Tong Wang, Miao Yu, Jianping Xu

**Affiliations:** Department of Endocrinology, Key Laboratory of Endocrinology, Ministry of Health, Peking Union Medical College Hospital, Diabetes Research Center of Chinese Academy of Medical Sciences & Peking Union Medical College, Beijing 100730, China; zhengjiapumc@163.com (J.Z.); rubiacordifolia@yahoo.com (Q.Z.); tongtong0716@sina.com (T.W.); yumiao@mendmail.com.cn (M.Y.); jpxuxh@163.com (J.X.)

**Keywords:** maternal low-protein diet, metabolic health, microRNAs, inflammation, early life, offspring

## Abstract

Emerging studies revealed that maternal protein restriction was associated with increased risk of type 2 diabetes mellitus in adulthood. However, the mechanisms of its effects on offspring, especially during early life of offspring, are poorly understood. Here, it is hypothesized that impaired metabolic health in offspring from maternal low-protein diet (LPD) is associated with perturbed miRNAs expression in offspring as early as the weaning age. We examined the metabolic effects on the C57BL/6J mice male offspring at weaning from dams fed with LPD or normal chow diet (NCD) throughout pregnancy and lactation. Maternal LPD feeding impaired metabolic health in offspring. Microarray profiling indicated that mmu-miR-615, mmu-miR-124, mmu-miR-376b, and mmu-let-7e were significantly downregulated, while, mmu-miR-708 and mmu-miR-879 were upregulated in LPD offspring. Bioinformatic analysis showed target genes were mapped to inflammatory-related pathways. Serum tumor necrosis factor-α (TNF-α) levels were higher and interleukin 6 (IL-6) had a tendency to be elevated in the LPD group. Finally, both mRNA and protein levels of IL-6 and TNF-α were significantly increased in the LPD group. Our findings provide novel evidence that maternal LPD can regulate miRNAs expression, which may be associated with chronic inflammation status and metabolic health in offspring as early as the weaning age.

## 1. Introduction

The prevalence of type 2 diabetes mellitus (T2DM) is increasing significantly throughout the world. However, the pathogenesis of diabetes has not been clearly demonstrated. Traditionally, it is generally believed that genes and adult lifestyle are critical factors of some metabolic diseases. Recently, several epidemiological studies of human populations highlighted that maternal nutrition, including over-nutrition and under-nutrition, may strongly influence the risks of developing metabolic diseases in adult life [1,2,3]. The mechanism underlying the increased susceptibility of T2DM in offspring of maternal malnutrition is poorly determined. 

Recently, it seems as though epigenetic modifications may be one of the major mechanisms explaining the association between early life malnutrition and late-onset diseases, such as obesity, insulin resistance, impaired glucose tolerance, and T2DM [4,5,6,7]. MicroRNAs (miRNAs) have emerged as important epigenetic modifications in recent years. They are a major class of small non-coding RNAs with about 20–22 nucleotides, which can mediate posttranscriptional regulation of gene expressions. miRNAs can specifically bind to the 3′-untranslated regions (3′-UTR) of target genes, resulting in translation inhibition [8]. Numerous studies show that miRNAs play critical roles in the pathogenesis of diabetes, such as glucose uptake, transport, and insulin secretion [9]. 

Human studies have revealed that poor intrauterine environment elicited by maternal dietary insufficiency or imbalance increased the risk for intrauterine growth restriction (IUGR) and led to metabolic diseases in the offspring during adulthood, such as insulin resistance, impaired glucose tolerance, and T2DM [3,10]. The maternal protein restriction model, with 5%–9% protein, as compared to 20% protein in normal diet, has been one of the most extensively studied models [11]. However, these studies were confined to long-term influences in offspring of a maternal low-protein diet (LPD) with varying durations range from 1 to 18 months (adulthood to aging) [12,13,14], yet the metabolic health in the early life of offspring, such as at weaning, has not been well documented. Furthermore, little information is known about the role of miRNAs between maternal low-protein diet (LPD) and glucose metabolism in the early life of offspring, such as at weaning. It is noted that computational predictions of miRNA targets based on several databases exhibits that one single miRNA can affect a gamut of different genes, ranging from several to hundreds, suggesting that a large proportion of the transcriptome is subjected to miRNA modulation [15]. Thus, using a trans-generational mouse model of maternal protein restriction and microarray platform, we address the hypothesis that impaired metabolic health in offspring from LPD-fed dams is associated with perturbation of the programmed expression of key miRNAs in offspring as early as at weaning age.

## 2. Materials and Methods

### 2.1. Animals and Diets

The seven-week-old C57BL/6J female and male mice were purchased from the Institute of Laboratory Animal Science, Chinese Academy of Medical Sciences and Peking Union Medical College (Beijing, China). All of the mice were maintained under controlled conditions with room temperature at 22 ± 2 °C and 12-h light/dark cycle. The mice were fed a normal chow diet (NCD) (D02041001, Research Diets, New Brunswick, NJ, USA) for one week for acclimatization. Then, mating was performed by housing males with females together (male:female = 1:2) until a vaginal plug was detected, which was considered day 0.5 of pregnancy. Pregnant females were single-housed and were randomly assigned to either isocaloric LPD (9.6% protein, 80.2% carbohydrate, and 10.2% fat as kcal%, D02041002, Research Diets, New Brunswick, NJ, USA) or NCD (23.5% protein, 66.3% carbohydrate, and 10.2% fat as kcal%, D02041001, Research Diets, New Brunswick, NJ, USA) during pregnancy and lactation. Thus, offspring were divided into two groups according to maternal diets as the LPD group and the NCD group. At day 1 after birth, all of the litters were adjusted to six pups each to ensure no litter was nutritionally biased. Birth weight was measured by calculating the average of one litter. All offspring were weaned at 21 days postnatal. At weaning, only one male offspring was randomly selected from one litter and was sacrificed in each group (one mouse per litter, *n* = 6 to 8 per group). The female offspring were not examined in our present study in order to prevent confounding factors related to their hormone profile and estrus cycle. Blood samples were taken from the intraorbital retrobulbar plexus after 12-h of fasting in anesthetized mice, and the liver samples were quickly removed, snap frozen in liquid nitrogen, and stored at −80 °C for further analysis. Body weight in offspring and food intake of dams were monitored weekly. All of the animal experiments were conducted in accordance with the Guide of the Care and Use of Laboratory Animals (NIH Publication No. 86-23, revised 1996) and were approved by the Animal Care and Use Committee of the Peking Union Medical College Hospital (Beijing, China, MC-07-6004).

### 2.2. Glucose Tolerance Tests

The tolerance test was performed as described previously [16]. Mice were overnight-fasted (12–16 h) and fasted blood glucose was measured in tail vein blood samples. Mice were injected intraperitoneally with glucose (2 g/kg body weight), and blood glucose was measured at 30 min, 60 min, and 120 min following injection using a glucometer (Bayer, Beijing, China). Blood glucose response to glucose tolerance tests was calculated as the area under the glucose curve for each mouse according to the trapezoidal method, as previously described [17].

### 2.3. Measurement of Serum Insulin and Inflammatory Factors

Serum insulin levels were measured using the mouse ultrasensitive insulin enzyme-linked immunosorbent assay (ELISA) kit (80-INSMSU-E01, ALPCO Diagnostics, Salem, NH, USA). Serum interleukin 6 (IL-6) and tumor necrosis factor-α (TNF-α) concentrations were measured by mouse ELISA kits (ab100712 and ab108910, Abcam, MA, USA), respectively.

### 2.4. Microarray Profiling of MiRNAs in Offspring

Because of financial constraints, we could not perform a whole genome array for each mouse in the LPD and NCD groups. Thus, in order to obtain a relatively reliable estimate of the mean gene expression, each group contained three biological replicates, which were randomly selected from each group. We performed the microarray with pooled RNA samples, a method that has been shown to be appropriate and statistically valid for efficient microarray experiments, according to previous studies [18,19]. As our study previously described [20], total RNA was extracted from the liver tissues in LPD and NCD offspring using Trizol reagent (Life Technologies Inc., Carlsbad, CA, USA), according to the manufacturer’s instructions. MiRNAs expressions in livers were detected by GeneChip microRNA 3.0 Array (Affymetrix, Inc., Santa, CA, USA), which provides for 100% miRBase v17 coverage [21,22].

### 2.5. Differential MiRNAs Expression Analysis in Offspring

Robust Multi-array Analysis (RMA) was utilized to convert raw data into recognizable miRNA expression data. Then it was followed by median normalization and log_2_ transformation using Affy package [23]. Differentially-expressed miRNAs between the LPD and NCD groups were analyzed by the Limma package of R language [23], which is based on the combined two criteria for true positive differences: (1) |FC (fold change)| ≥ 2 and (2) *p* value < 0.05 [21], which is a relatively robust cutoff point.

### 2.6. Bioinformatics Analysis of Predicted Targets for MiRNAs in Offspring

All of the microarray data were also pooled for further analysis. According to our previously published work [24], target genes of differentially expressed miRNAs between the two groups were identified using the miRWalk database [25], which can provide validated target genes information on miRNAs for mouse [26]. To further reveal the potential biological functions and pathways of the target genes, the target genes were analyzed with the Kyoto Encyclopedia of Genes and Genomes (KEGG) pathways [27] using Database for Annotation, Visualization and Integrated Discovery (DAVID) [28]. *p* value < 0.05 was the criterion for significant KEGG pathway terms.

### 2.7. Validation of Differentially Expressed MiRNAs in Offspring

In order to validate the expressions of differential miRNAs, quantitative real-time PCR (qRT-PCR) were utilized to detect the relative expression of differentially-expressed miRNAs, with samples enlarged in each group (*n* = 6 to 8 per group) using the TaqMan detection system (Life Technologies, Foster City, CA, USA). All of the mice in each group were included for the validation. Total RNA was reversely transcribed using the TaqMan MicroRNA Reverse Transcription kit (Applied Biosystems, Life Technologies, Foster City, CA, USA). All of the miRNA-specific reverse-transcription primers were provided with the TaqMan MicroRNA Assay and purchased from Life Technologies Corporation (Applied Biosystems, Life Technologies, Foster City, CA, USA). The primers were mmu-miR-615 (ID 2353), mmu-miR-124 (ID 2197), mmu-miR-376b (ID 2452), mmu-let-7e (ID 2407), mmu-miR-708 (ID 1643), and mmu-miR-879 (ID 2473). U6 small nuclear RNA (ID 1973) was used as an endogenous control. Gene expression was quantified by qRT-PCR and performed on an ABI prism Vii7 Sequence Detection System platform (ABI Prism^®^ Vii7, Applied Biosystems, Life Technologies, Foster City, CA, USA). Data were analyzed and the fold change was calculated using the comparative Ct method. All reactions were carried out with three biological replicates, and each analysis consisted of three technical replicates.

### 2.8. Target Gene Expression by Quantitative Real-Time PCR

For the validation of miRNA target genes, mRNA expressions of target genes were performed using SYBR Green. Prior to PCR, total RNA of each sample was processed with Rnase-free Dnase (Qiagen, New York, NY, USA). The RNA was reverse transcribed by 1 μg of total RNA from each sample using the Power cDNA Synthesis kit (A3500, Promega BioSciences LLC, Sunnyvale, CA, USA). We used Oligo 7.0 software (Molecular Biology Insights, Inc., Cascade, CO, USA) to design the sequences of the primers for IL-6, TNF-α, and housekeeping gene β-actin. The sequences of the primers are as following: IL-6, forward 5′-CCAAGAGGTGAGTGCTTCCC-3′, reverse 5′-CTGTTGTTCAGACTCTCTCCCT-3′; TNF-α, forward 5′-CCCACGTCGTAGCAAACCA-3′, reverse 5′-ACAAGGTACAACCCATCGGC-3′; MAPK1, forward 5′-AATTGGTCAGGACAAGGGCT-3′, reverse 5′-GAGTGGGTAAGCTGAGACGG-3′; β-actin, forward 5′-TGTTACCAACTGGGACGACA-3′ reverse 5′-GGGGTGTTGAAGGTCTCAAA-3′. The reaction production was accurately measured by the ABI prism Vii7 Sequence Detection System (ABI Prism^®^ Vii7, Applied Biosystems, Life Technologies, Foster City, CA, USA). Data were analyzed and quantified using the comparative Ct method, as described in the preceding section.

### 2.9. Immunohistochemical Staining

The liver tissues from offspring mice were rapidly dissected. Then, they were fixed overnight in a freshly-prepared 10% buffered formaldehyde solution. The tissue samples were ethanol-dehydrated and embedded in paraffin wax. Serial 5 µm paraffin embedded tissue sections were mounted on slides. Then, the slides were stained with the primary antibodies overnight at 4 °C, which were IL-6 (1:300; ab83339, Abcam, Cambridge, MA, USA) and TNF-α (1:50, ab6671, Abcam, Cambridge, MA, USA), while negative controls were incubated with phosphate-buffered saline (PBS). The slices were then washed and incubated for 1 h at 37 °C with secondary antibodies (1:1000, Cell Signaling, Danvers, MA, USA). Then, the slides were stained with 3, 3′-diaminobenzidine (DAB) and hematoxylin. Brownish yellow granular or linear deposits were identified as positive. Image-Pro Plus 5.0 (Media Cybernetics, Silver Spring, MD, USA) was used for semi-quantitative analysis.

### 2.10. Statistical Analysis

Results are expressed as the mean ± SEM. Statistical analyses were performed with Student’s *t*-tests of unpaired samples. The comparisons of glucose tolerance tests were analyzed with a two-way ANOVA. Fisher’s exact test was used for KEGG pathway analysis. A *p* value < 0.05 was considered statistically significant. Prism version 6.0 (GraphPad Software Inc., San Diego, CA, USA) was used for statistical analysis.

## 3. Results

### 3.1. Effects of Diets on Body Weight and Glucose Tolerance in Dams

**Figure 1:**
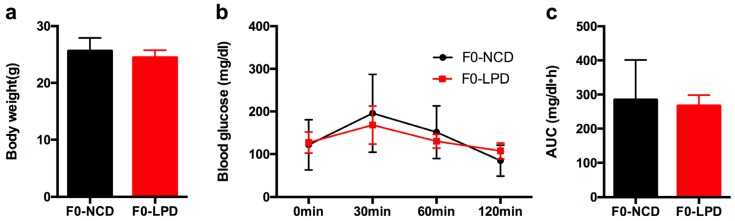
Effects of diets during pregnancy and lactation on metabolic phenotype in dams. (**a**) Body weight; (**b**) intraperitoneal glucose tolerance tests; and (**c**) area under the curve (AUC) of glucose tolerance tests. Data represented as the mean ± SEM (*n* = 6 to 8, per group). Dams were noted as F0; LPD, low-protein diet; NCD, normal chow diet.

**Figure 2:**
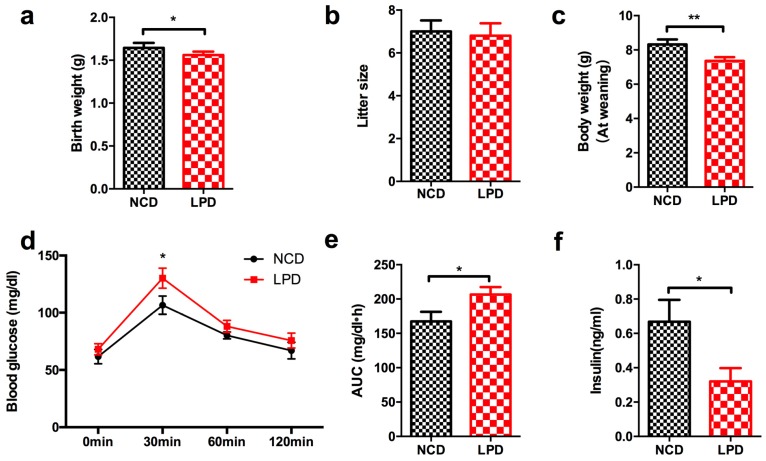
Metabolic profile and serum insulin concentration in offspring at weaning. (**a**) Birth weight; (**b**) litter size; (**c**) body weight at weaning; (**d**) intraperitoneal glucose tolerance test; (**e**) area under curve (AUC) of glucose tolerance test; and (**f**) serum insulin level. Data represented as the mean ± SEM (*n* = 6 to 8, per group). * *p* < 0.05, ** *p* < 0.01 vs. the NCD group. LPD, low-protein diet; NCD, normal chow diet.

By the end of lactation, there was no significant difference in maternal body weight between the dams (noted as F0) fed with LPD and NCD (Figure 1a). Glucose tolerance testing showed that blood glucose was indistinguishable between the two groups, as well as with the area under the curve (AUC) (Figure 2b,c). There was no significant difference in food intake between dams fed with LPD and NCD.

### 3.2. Effects of Maternal Diet on Metabolic Profile in Offspring at Weaning

At birth, newborns whose mother was fed with LPD during pregnancy and lactation showed lower body weight compared to offspring of control diet mothers (*p* < 0.05, Figure 2a). No significant difference was found in litter size between LPD and NCD groups (Figure 2b). At weaning, the male offspring of LPD-fed dams exhibited significantly lower body weight, compared with the offspring of dams fed a control diet (*p* < 0.05, Figure 2c). There was no difference of fasted blood glucose in the LPD and NCD male offspring at weaning. However, the blood glucose of the male offspring in LPD group was higher at 30 min (*p* < 0.05) after intraperitoneal glucose administration (Figure 2d). Consistently, the area under the curve (AUC) of the glucose tolerance test was greater in LPD offspring (*p* < 0.05, Figure 2e). We further examined insulin concentration of the male offspring. As consistent with previous studies [15,29], LPD offspring showed lower fasted insulinemia at weaning, compared to control offspring (Figure 2f), which may be caused by aberrant insulin secretion due to intrauterine growth restriction.

### 3.3. Differential MiRNAs Expression in Offspring

**Figure 3:**
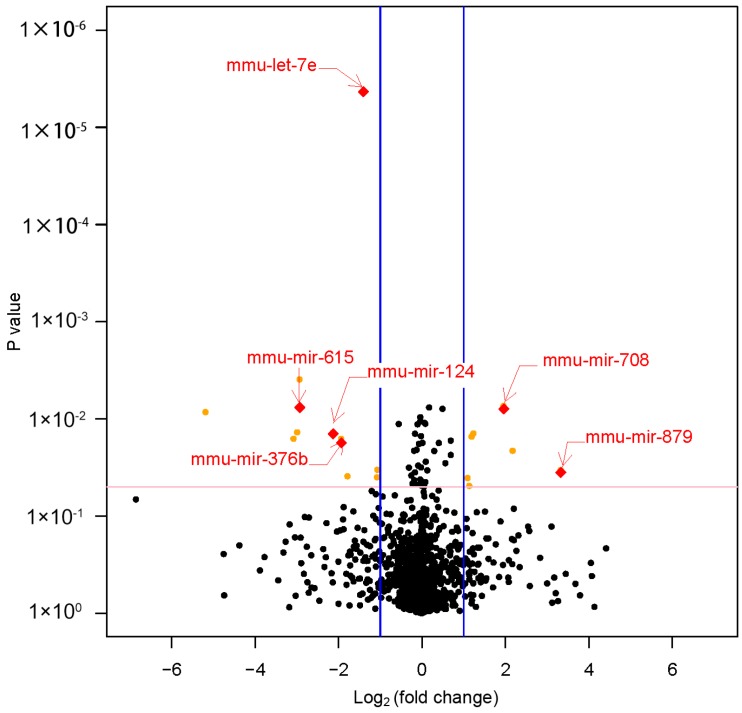
The volcano plot of the miRNA array. This graph shows log_2_ (fold change) in the expression miRNAs and *p* value from the t-test between LPD and NCD offspring. The vertical blue line indicates that the threshold of |log_2_ (fold change)| is 1. The horizontal red line indicates that the *p* value threshold is 0.05. It showed six miRNAs were significantly differentially expressed (|fold change| ≥ 2 and *p* value < 0.05) between LPD and NCD offspring. LPD, Low-protein diet; NCD, normal chow diet.

Six miRNAs were shown to be significantly differentially expressed (|fold change| ≥ 2 and *p* value < 0.05) between LPD and NCD groups. In the livers of LPD offspring, mmu-miR-615, mmu-miR-124, mmu-miR-376b, and mmu-let-7e were significantly downregulated (fold change ≤ −2 and *p* value < 0.05). Meanwhile, mmu-miR-708 and mmu-miR-879 were significantly upregulated (fold change ≥ 2 and *p* value < 0.05) (Table 1, Figure 3).

### 3.4. Validation of Differentially Expressed MiRNAs

**Figure 4:**
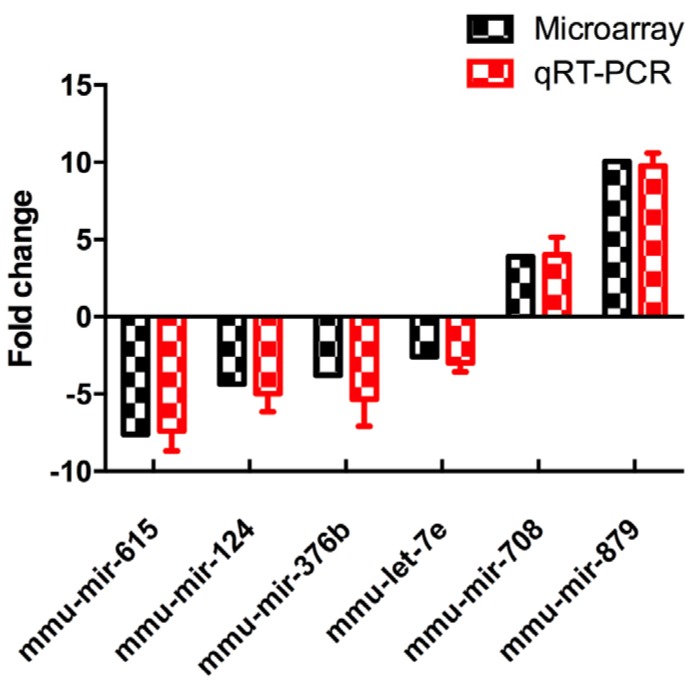
Differentially-expressed miRNAs were detected by miRNA array and validated by qRT-PCR. Data represented as the mean ± SEM (*n* = 6 to 8, per group). The fold change was calculated using the comparative Ct method. qRT-PCR: quantitative real time-PCR.

To verify the results of microRNA array, all the six differentially-expressed miRNAs were validated by increasing the quantity of samples in LPD and NCD groups using qRT-PCR (*n* = 6 to 8, per group). It showed that the expression levels of all the six miRNA were consistent with the results of microRNA array analysis, which were analyzed and quantified by qRT-PCR (Figure 4).

### 3.5. Functional Enrichment Analysis for Target Genes by Bioinformatics Analysis

Furthermore, target genes of these six differentially-expressed miRNAs were identified by the miRWalk database. The six miRNAs, including mmu-miR-615, mmu-miR-124, mmu-miR-376b, mmu-let-7e, mmu-miR-708, and mmu-miR-879 had a total of 349 validated target genes in the miRWalk database (Table 2). By utilizing the Database for Annotation, Visualization and Integrated Discovery (DAVID) and Kyoto Encyclopedia of Genes and Genomes (KEGG) databases, the functional enrichment analysis of these 349 validated genes were mapped in the MAPK signaling pathway, TGF-beta signaling pathway, Jak-STAT signaling pathway, cytokine-cytokine receptor interaction, chemokine signaling pathway, adipocytokine signaling pathway, and Toll-like receptor signaling pathway (Table 3), which are all associated with inflammation responses.

### 3.6. Effects of Maternal Diet on Serum Pro-Inflammatory Cytokines in Offspring

**Figure 5:**
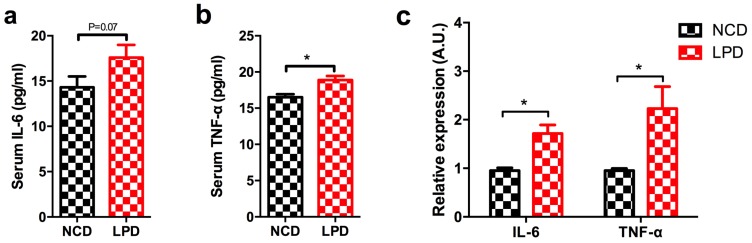
Effect of maternal LPD on serum IL-6 and TNF-α concentrations and mRNA expression in the offspring at weaning. (**a**) Serum IL-6; (**b**) serum TNF-α; and (**c**) IL-6 and TNF-α mRNA expression in the livers of offspring. Data represented as the mean ± SEM (*n* = 6 to 8, per group). * *p* < 0.05, vs. the NCD group. LPD, low-protein diet; NCD, normal chow diet; IL-6: interleukin-6; TNF-α: tumor necrosis factor-α.

Our functional enrichment analysis indicated that target genes of our six differentially-expressed miRNAs were mapped in inflammatory pathways (Table 2 and Table 3). *IL-6* and *Tnf* were two important target genes, which were located in several pathways (marked as bold in Table 3). Therefore, we first measured two important pro-inflammatory cytokines (serum IL-6 and TNF-α) in male offspring of dams fed with LPD and NCD, to determine whether maternal diets modulated the levels of inflammatory markers in the early life of offspring. It showed there was a tendency to be higher of serum IL-6 level in LPD male offspring (*p* = 0.07) (Figure 5a), and it also showed the level of serum TNF-α was significantly elevated in LPD male offspring at weaning (*p* < 0.05) (Figure 5b). However, no difference of MAPK1 expression was observed in offspring (data not shown).

### 3.7. Differential Expression of Pro-Inflammatory Markers in Offspring

**Figure 6:**
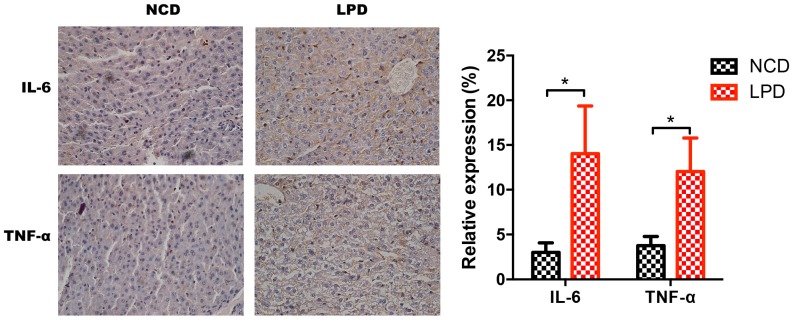
Immunohistochemistry for *IL-6* and *TNF-α* expression and semi-quantitative assessments. (**left**) *IL-6* and *TNF-α* staining in NCD and LPD groups. (**right**) Semi-quantitative scores of *IL-6* and *TNF-α*. Data represented as the mean ± SEM (*n* = 6 to 8, per group). * *p* < 0.05, vs. the NCD group. LPD, low-protein diet; NCD, normal chow diet; IL-6: interleukin-6; TNF-α: tumor necrosis factor-α.

Next, we measured *Il-6* and *TNF-α* expression in liver samples of male offspring of LPD- and NCD-fed dams using qRT-PCR. It indicated that *IL-6* and *TNF-α* expression were significantly increased in the LPD group, and the fold change of IL-6 and TNF-α expression is about 1.8 and 2.2, respectively (*p* < 0.05, Figure 5c). To further determine whether the protein expressions of *IL-6* and *TNF-α* in offspring were modulated by maternal LPD, the protein expressions of these two genes were examined by immunohistochemistry. As shown in Figure 6, there was a statistically significant increase in the immunoreactivities of *IL-6* and *TNF-α* in the early life of offspring exposed to maternal LPD during pregnancy and lactation, compared to offspring of NCD-fed dams (*p* < 0.05).

## 4. Discussion

**Figure 7:**
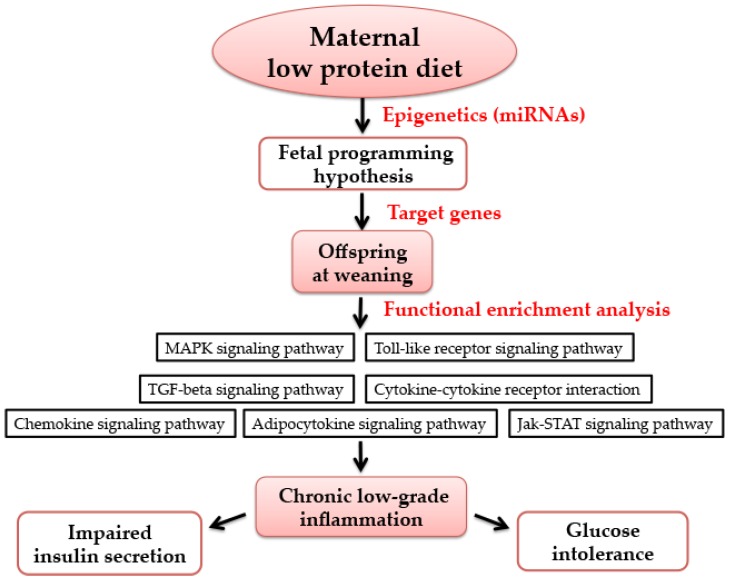
The possible mechanism of maternal low-protein diet during pregnancy and lactation on offspring at weaning. Maternal nutrition has long-term metabolic effects on offspring, which is known as the “fetal programming hypothesis”. Epigenetics (such as miRNAs) has been deemed as an important molecular basis of maternal nutrition and metabolic health in offspring. Using functional enrichment analysis, the target genes of differentially-expressed miRNAs were mapped into seven pathways, which are all associated with inflammation. Thus, it may be related to chronic low-grade inflammation, which may cause impaired insulin secretion and glucose intolerance. MAPK: mitogen-activated protein kinase; TGF: transforming growth factor; Jak-STAT: Janus kinase-signal transducer and activator of transcription.

Epidemiological studies and animal experiments have indicated that adverse maternal nutriture influence organ development and metabolism of progeny, which may increase the susceptibility of developing diabetes in later life, and even in the following generation [30,31]. Maternal nutrition has long-term metabolic effects on offspring, which was known as the “fetal programming hypothesis” [32]. Here, our findings demonstrate that the exposure of LPD feeding during pregnancy and lactation resulted in low birth weight, impaired glucose tolerance, and lower insulin secretion in offspring, at as early as weaning age. We also observed that offspring from LPD-fed dams showed perturbation of the programmed expression of some key miRNAs in offspring at weaning. Thus, using microarray profiling, bioinformatics approaches, and functional enrichment analysis, all of the target genes of misexpressed miRNAs were mapped in seven inflammatory-related pathways. Finally, we showed that that serum levels of pro-inflammatory cytokines IL-6 and TNF-α were also increased in offspring from LPD-fed dams. Furthermore, the mRNA expression and protein levels of IL-6 and TNF-α were significantly increased in offspring from maternal LPD feeding. Therefore, these results show that maternal LPD consumption may regulate the expression of miRNAs, which may be associated with chronic inflammation status and glucose intolerance in offspring as early as weaning age (Figure 7).

Obesity, insulin resistance, and diabetes mellitus are associated with chronic low-grade inflammation [33,34,35]. One recent review summarized that chronic inflammation is characterized by increased levels of pro-inflammatory cytokines in response to physiological and environmental stimuli that can arrest the immune system in a low-level activation state [36]. Our study demonstrates that maternal protein restriction diet during pregnancy and lactation may induce an inflammatory state in offspring at weaning age. Similar to our study, one recent study showed that male Wistar rats exposed to a low-protein (8%) diet in utero that had a low birth weight, but then underwent postnatal catch-up growth exhibited higher indexes of inflammatory, with increased hepatic expression of interleukin-6 (IL-6) levels, tumor necrosis factor α (TNF-α), and monocyte chemotactic protein-1 (MCP-1) at 12 months of age [37]. Sílvia et al. also showed that maternal low-protein (6%) diet during pregnancy and lactation showed increased transcription of nuclear factor kappa B (NF-κB) and IL-6, with reduced transcription of interleukin-6 (IL-10) (an anti-inflammatory cytokine) at 90 days of age in offspring [38]. However, the inflammatory state of offspring was examined at an adult or even older age in the aforementioned studies. Here our findings demonstrate that maternal LPD feeding during pregnancy and lactation can induce an inflammatory state in the offspring as early as weaning age. In our previous study, we found that a maternal high-calorie diet is associated with altered hepatic microRNA expression and impaired metabolic health in offspring at weaning age [20]. The composition of the high-calorie diet is 16.4% protein, 25.6% carbohydrate, and 58% fat as the kcal%. However, we found that both of the two studies showed that maternal nutrition are associated with inflammation in offspring. We cannot draw a conclusion that the same biological mechanisms are at play in these two studies because our study is very preliminary, which needs further experimentation and validation. However, we can speculate that maternal malnutrition (whether over-nutrition or under-nutrition) may be associated with aberrant metabolic health and inflammation status in offspring at weaning.

Increasing evidence showed that epigenetic information could be inherited as a trans-generational carrier between generations. It showed that nutrient-dependent regulation of miRNAs may trigger disease susceptibility and metabolic complications in offspring [39]. One clinical study indicated that placental expression of miR-210 increased in preeclampsia and was inversely related to birth weight and gestational age [40]. It showed that a maternal low-protein diet exhibited impaired glucose tolerance and suppressed pancreatic β-cell proliferation in mouse offspring via miR-15b at 12 weeks of age [41]. In addition to maternal nutrition, the advanced fetal programming hypothesis proposes that maternal genetic variants may influence the offspring’s phenotype indirectly via epigenetic modification. Hocher et al. showed that altered expression of hepatic microRNA was observed in offspring of female eNOS −/+ mice, demonstrating that a maternal genetic defect can epigenetically alter the phenotype of the offspring, without inheritance of the defect itself [42]. Recently, inflammatory signaling has been identified as an epigenetic mediator and increasing evidence has shown the epigenetics can regulate inflammatory gene expression, which can ultimately result in multiple adverse physiological consequences [43]. Our study shows that validated target genes of differentially-expressed miRNAs in offspring from LPD-fed dams are part of inflammatory signaling pathways. *IL-6* and *TNF* are two validated target genes of let-7e. Recent findings offer a putative role of non-coding RNAs, especially miRNAs, in the progression and management of the inflammatory response [44]. The let-7 family of miRNAs is highly conserved across diverse animal species, and plays critical roles in the regulation of cell proliferation and differentiation [45]. In addition to cancer, an altered expression of let-7 has been reported in inflammation. Let-7 miRNAs demonstrated to be repressed in inflammation, which resulted in increased expression of pro-inflammatory cytokines and increased inflammatory status [46], which is consistent with our results. One clinical study showed that let-7e target genes were predicted to be associated with ulcerative colitis susceptibility [47]. In addition, several miRNAs, such as miR-126, miR-132, miR-146, miR-155, and miR-221 have also emerged as important transcriptional regulators of some inflammation-related mediators [44]. Therefore, we propose that maternal protein restriction diet induce altered hepatic microRNA expression, which may be associated with enhanced inflammatory responses in offspring as early as at weaning age.

To the best of our knowledge, our study provides the first evidence that maternal LPD feeding may regulate the expression of miRNA, which may be associated with chronic inflammation status and glucose intolerance in offspring as early as weaning age. However, some limitations should be considered in our study. First, we agree with the reviewer that our data is preliminary and cannot exclude the possibility that the association between miRNAs and markers of inflammation is due to a state of chronic systemic inflammation causing altered hepatic miRNA, rather than the other way around. Second, our present study focused on the mechanisms between maternal protein restriction and its detrimental effects on offspring during early life of offspring and there was no data on long-term effects in adulthood of our model. Our further research will be focused on the miRNAs expression and inflammation status in older mice offspring to examine the long-term effects in adulthood of our model. Third, the function of miRNA analysis, which showed to be related to inflammation, is mainly based on microarray profiling, bioinformatics analysis, and some preliminary molecular biology experiments. Therefore, using in vivo and in vitro experiments, further studies should be investigated to demonstrate the role of perturbed miRNA expression on glucose metabolism and inflammation status. A fourth limitation is that it is unknown when the critical time window of maternal LPD consumption is; during the gestation period or lactation period, or both periods. Our undergoing work aims to determine the effects of LPD in the dams on glucose metabolism in offspring in specific time window. Furthermore, recent reviews showed that paternal nutritional challenges can also cause programming of glucose impairment in the offspring and alterations of noncoding sperm micro-RNAs, histone acetylation, and targeted, as well as global, DNA methylation seem to be particularly involved in paternal programming of offspring’s diseases in later life [48,49]. Therefore, our future study will be interested in examining the underlying epigenetic mechanism linking paternal nutrition and its programming effect on offspring.

In conclusion, here we demonstrated that maternal protein restriction diet with IUGR predisposes to impaired glucose tolerance in the offspring as early as weaning age. This study contributes to the current knowledge on the putative role of miRNAs between maternal low-protein diet and metabolic health in offspring, which are likely to be associated with inflammatory responses. A better understanding of the function of these miRNAs might open the way to the development of new strategies for the early prevention of diabetes. Thus, further studies in the field of trans-generational effects to clarify the underlying mechanisms are urgently warranted.

## Figures and Tables

**Table 1:** Differentially-expressed miRNA (|fold change| ≥ 2 and *p*-value < 0.05).

Probe Set ID	Fold Change	*p* Value	Sequence Length	Sequence
**mmu-miR-615**	−7.61	0.004	22	GGGGGUCCCCGGUGCUCGGAUC
**mmu-miR-124**	−4.37	0.014	22	CGUGUUCACAGCGGACCUUGAU
**mmu-miR-376b**	−3.81	0.016	21	AUCAUAGAGGAACAUCCACUU
**mmu-let-7e**	−2.60	0.000	22	CUAUACGGCCUCCUAGCUUUCC
**mmu-miR-708**	3.89	0.007	23	AAGGAGCUUACAAUCUAGCUGGG
**mmu-miR-879**	10.05	0.034	22	GCUUAUGGCUUCAAGCUUUCGG

**Table 2:** Validated targeted genes for differentially-expressed miRNAs.

MiRNA	Count	Target Genes
**mmu-miR-615**	6	*Msx2*, *Hoxa7*, *Mbp*, *Lin28*, *Cdkn2a*, *Igf2*
**mmu-miR-124**	87	*Dll1*, *Sox9*, *Camk2g*, *Zic2*, *Fabp7*, *Hod*, *Pou5f1*, *Dicer1*, *Fgf8*, *Fgf10*, *H1foo*, *Bmp4*, *Lin28*, *Tcfap2b*, *Calb2*, *Btg2*, *Dcx*, *Pax6*, *Ifitm3*, *Dppa3*, *Mapk14*, *Evi1*, *Nr4a2*, *Mtpn*, *Nefm*, *Eomes*, *Cpeb1*, *Ctdspl*, *Mstn*, *Gata1*, *Stmn2*, *Rest*, *Trpm3*, *Mbp*, *Hprt1*, *Th*, *Oog4*, *Des*, *Uchl1*, *Foxp2*, *Rho*, *Eif2c3*, *Efnb1*, *Dnmt3b*, *Dbh ,H2afx*, *Npy*, *Med13*, *Prkca*, *Eif2c2*, *Fgf21*, *Phox2a*, *Mos*, *Gbx2*, *Emx1*, *Myh6*, *Mt1*, *Eif2c1*, *L1cam*, *Phox2b*, *Ctdsp1*, *Lmx1b*, *Tbr1*, *Nppa*, *Ccne1*, *Ccnb2*, *Eif2c4*, *Casp3*, *Tcfap2a*, *Sycp1*, *Gja1*, *Zp3*, *Rfpl4*, *Cdh1*, *Vax2*, *Slc6a3*, *Dlk1*, *Ntrk2*, *Pou3f3*, *Myh7*, *Sycp3*, *H2afz*, *Stat3*, *Wnt1*, *Foxa2*, *Ntrk3*, *Gja5*
**mmu-miR-376b**	28	*Hprt1*, *Oog4*, *Dnmt3b*, *H2afx*, *Fgf21*, *Mos*, *Dlk1*, *Mt1*, *Mbp*, *Ccne1*, *Ccnb2*, *Lin28*, *Zp3*, *Rfpl4*, *Gpr172b*, *Sycp3*, *H2afz*, *Timp4*, *Camk2g*, *Dicer1*, *Atg4c*, *Pou5f1*, *H1foo*, *Frap1*, *Ifitm3*, *Dppa3*, *Cpeb1*, *Ctdspl*
**mmu-let-7e**	217	*Lin28*, *Casp3*, *Ptch1*, *Zfp106*, *Mov10*, *Fgf16*, *E2f6*, *Fgf21*, *Ptges*, *Akt1*, *Scpep1*, *Rad52*, *Mgst1*, *Cdc34*, *Git1*, *Ebp*, *Mos*, *Kras*, *Il6*, *Capn10*, *Col1a1*, *Smad4*, *Hace1*, *Igfbp3*, *Fas*, *Hmga2*, *Fgfr1*, *Sox2*, *Irf4*, *Mt1*, *Kcnj16*, *Socs1*, *Mmp9*, *Prl*, *Nr2e1*, *Sp1*, *Il10*, *Trim71*, *Notch1*, *Nrip1*, *Lamc1*, *Acvr2a*, *Smox*, *Igf2*, *Cdkn1a*, *Egr1*, *Ccne1*, *Tagln*, *Akt1*, *Ccnb2*, *Vsnl1*, *Spp1*, *Egfr*, *Syne1*, *Mdk*, *Hnf4a*, *Msi1*, *Dnmt3a*, *Cd4*, *Sparcl1*, *Hspd1*, *Tppp3*, *Vim*, *Smad3*, *Tnf*, *Cyp2b10*, *Gtf2h4*, *Zp3*, *Hyou1*, *E2f2*, *Csf1r*, *Rfpl4*, *Akap6*, *Stat3*, *Golph3*, *Snai1*, *Arc*, *Scamp2*, *bp1*, *Mov10*, *Nanog*, *Clock*, *Acvr1*, *Wnt1*, *Bcl2l1*, *BC060632*, *Cdkn2a*, *Trp53*, *Fn1*, *Sycp3*, *Tpm1*, *Scpep1*, *Lpar1*, *H2afz*, *Gmfb*, *Pten*, *Arc*, *Snai1*, *Dync1i1*, *Mapre1*, *Dcn*, *Bcl2*, *Scpep1*, *Hras1*, *Igf1*, *E2f6*, *Grb2*, *Camk2g*, *Ogt*, *Pgc*, *Ctcf*, *Dicer1*, *Mapk1*, *Scpep1*, *Cxcr4*, *Rcan1*, *Clu*, *Dut*, *Piwil2*, *Ifng*, *Mtpn*, *Dclk1*, *Igfbp3*, , *Igf1r*, *Akt1*, *Kdr*, *Nanog*, *Hras1*, *Neurod1*, *Dppa3*, *H1foo*, *Bmpr2*, *Ebp*, *Hmox1*, *Gpd1*, *Cyp2b10*, *Socs3*, *Inha*, *Eif2c2*, *Epb4.1l3*, *Serpina1c*, *Ssr3*, *E2f2*, *Dicer1*, *Mstn*, *Pten*, *Ifitm3*, *Dhcr24*, *Racgap1*, *Dppa3*, *Gad1*, *Ccr4*, *Ddit3*, *Nr4a1*, *Hmga1*, *Ppargc1a*, *Bcl2*, *Nr6a1*, *Gadd45a*, *Scpep1*, *Ptp4a2*, *Nanog*, *Runx2*, *Zeb1*, *Bak1*, *Ghr*, *Birc2*, *Sall4*, *Cpeb1*, *Capn8*, *Il23r*, *Ctdspl*, *Ptges3*, *Ephb2*, *Rpe*, *Syt4*, *Trim32*, *Foxp1*, *Scpep1*, *Mmp14*, *Bcl2*, *Gnb1*, *Madd*, *Pgc*, *Gnrh1*, *Hmox1*, *Myc*, *Mycn*, *Socs1*, *Jarid1b*, *Hprt1*, *Cyp2b10*, *Oog4*, *Pdzd7*, *Cdkn1a*, *Cebpb*, *Aqp4*, *Rdx*, *Mbp*, *Hand1*, *Bcl2*, *Lancl1*, *Mapk3*, *Casp9*, *Fmr1*, *Klf15*, *Dnmt3b*, *Adora1*, *Stx1a*, *Kitl*, *Cd34*, *H2afx*, *Itgb1*, *Smad5*, *Pou5f1*
**mmu-miR-708**	9	*Foxo3*, *Cd34*, *Mbp*, *Lin28*, *Stat5a*, *Cd34*, *E2f6*, *Aqp1*, *Bmi1*
**mmu-miR-879**	2	*Mbp*, *Lin28*

**Table 3:** Validated target genes enriched by the Kyoto Encyclopedia of Genes and Genomes (KEGG) pathway.

KEGG ID	Term	Count	%	*p* Value	Genes
**mmu04010**	MAPK signaling pathway	24	9.7	2.8 × 10^−8^	*Egfr*, *Prkca*, *Trp53*, *Fgfr1*, *Fgf8*, ***Tnf***, *Grb2*, *Fgf16*, *Fgf10*, *Nr4a1*, *Fgf21*, *Ddit3*, *Hras1*, *Akt1*, *Mapk1*, *Casp3*, *Kras*, *Mapk14*, *Ntrk2*, *Mapk3*, *Mos*, *Fas*, *Myc*, *Gadd45a*
**mmu04350**	TGF-beta signaling pathway	14	5.7	7.3 × 10^−8^	*Bmp4*, ***Tnf***, *Smad5*, *Bmpr2*, *Smad4*, *Smad3*, *Dcn*, *Mapk1*, *Acvr2a*, *Sp1*, *Ifng*, *Mapk3*, *Myc*, *Acvr1*
**mmu04630**	Jak-STAT signaling pathway	14	5.7	4.4 × 10^−5^	***Il6***, *Il23r*, *Socs3*, *Grb2*, *Stat5a*, *Socs1*, *Bcl2l1*, *Stat3*, *Il10*, *Akt1*, *Ifng*, *Myc*, *Prl*, *Ghr*
**mmu04060**	Cytokine-cytokine receptor interaction	17	6.9	1.4 × 10^−4^	*Egfr*, ***Il6***, ***Tnf***, *Il23r*, *Bmpr2*, *Kitl*, *Il10*, *Kdr*, *Acvr2a*, *Ccr4*, *Cxcr4*, *Ifng*, *Fas*, *Prl*, *Acvr1*, *Ghr*, *Csf1r*
**mmu04062**	Chemokine signaling pathway	11	4.5	9.3 × 10^−3^	*Akt1*, *Mapk1*, *Kras*, *Ccr4*, *Gnb1*, *Cxcr4*, *Grb2*, *Mapk3*, *Foxo3*, *Stat3*, *Hras1*
**mmu04920**	Adipocytokine signaling pathway	6	2.4	1.9 × 10^−2^	*Akt1*, ***Tnf***, *Npy*, *Socs3*, *Ppargc1a*, *Stat3*
**mmu04620**	Toll-like receptor signaling pathway	7	2.8	2.7 × 10^−2^	*Akt1*, *Mapk1*, ***Il6***, ***Tnf***, *Mapk14*, *Mapk3*, *Spp1*

MAPK: mitogen-activated protein kinase; TGF: transforming growth factor; Jak-STAT: janus kinase-signal transducer and activator of transcription. *Il-6* and *Tnf* were marked as bold.
